# Peri-operative antibiotic prophylaxis in obstetrics: a 6-year evaluation of guideline adherence and stewardship impact in a Ukrainian maternity hospital

**DOI:** 10.1016/j.infpip.2026.100515

**Published:** 2026-02-10

**Authors:** O. Pechak, K. Bielka, M. Frank, H. Fomina, D. Yevstifeiev, L. Yanitska

**Affiliations:** a Department of Medical Biochemistry and Molecular Biology, Bogomolets National Medical University, Kyiv, Ukraine; b Department of Surgery, Anesthesiology and Intensive Care of Postgraduate Education, Bogomolets National Medical University, Kyiv, Ukraine

**Keywords:** peri-operative antibiotic prophylaxis, obstetric surgery, antimicrobial stewardship, guideline adherence, Ukraine, hospitalization

## Abstract

**Background:**

Surgical-site infections are a major concern in obstetric surgery. In Ukraine, evolving national guidelines and institutional antimicrobial stewardship (AMS) initiatives have sought to optimize peri-operative antibiotic prophylaxis (PAP), but their impact on clinical outcomes requires rigorous evaluation.

**Methods:**

We conducted a retrospective cohort study (2018–2023) of 474 obstetric surgeries to evaluate changes in PAP adherence and its association with length of stay (LOS). Adherence was assessed using a 3-point compliance score (timing, choice, and duration). The association between compliance and time to discharge was analysed using a multi-variable Cox proportional hazards model.

**Findings:**

A marked improvement in practice was observed; full compliance increased from 11% to 71%, while mean LOS decreased from 9.9 to 4.7 days. Component analysis showed that structural interventions eliminated duration errors, while errors in antibiotic choice persisted. In the multi-variable model, the ‘year of surgery’ was the strongest predictor of earlier discharge (*P* < 0.001), acting as a proxy for holistic systemic improvements. While the compliance score was not an independent predictor in the overall cohort, binary compliance showed a trend towards significance (*P* = 0.098), remaining statistically subordinate to secular trends. A significant association was found only in the highly standardized laparoscopy subgroup, suggesting the impact of protocol adherence is most visible in predictable clinical environments.

**Conclusion:**

Multi-faceted AMS programs can successfully optimize PAP adherence and improve outcomes. While the overall reduction in LOS is driven by broad improvements over time, specific guideline adherence remains a crucial quality indicator, particularly for standardized procedures.

## Introduction

Surgical-site infections (SSIs) remain among the most prevalent healthcare-associated infections globally, representing a leading cause of postoperative morbidity, prolonged hospitalization, and increased healthcare costs [1]. In obstetric surgery, peri-operative antibiotic prophylaxis (PAP) plays a critical role in reducing SSI risk and improving maternal outcomes [2]. International consensus guidelines emphasize that effective PAP requires three key elements: administration of an appropriate antibiotic within a defined preoperative window, avoidance of unnecessary postoperative continuation, and selection of agents aligned with local resistance patterns [3].

In Ukraine, regulatory standards for surgical prophylaxis have evolved significantly. The original national guideline (Ministry of Health [MOH] Order No. 502, 2008) was updated in 2022 under Order No. 822, which narrowed the optimal timing window to 30–60 min pre-incision and reinforced cefazolin as the preferred agent [4,5]. Concurrently, our institution, a tertiary referral centre, initiated a comprehensive antimicrobial stewardship (AMS) program in 2019 to bridge the gap between outdated national norms and international best practices.

This study addresses a critical knowledge gap by quantifying the impact of these concurrent regulatory and institutional interventions on both process measures (adherence) and key clinical outcomes (length of stay [LOS]). We aimed to evaluate adherence to modern PAP standards over a six-year period and to analyse the association between the level of adherence and patient LOS, using a rigorous analytical approach to account for potential confounding factors.

## Methods

We conducted a retrospective cohort study at a tertiary maternity hospital in Kyiv, Ukraine. The study included 474 women who underwent hysteroscopy, laparoscopy, or laparotomy between January 2018 and December 2023. Data were extracted from electronic medical records, anaesthesia protocols, and pharmacy dispensing logs using a standardized case report form, which demonstrated excellent inter-rater reliability (κ = 0.92) during pilot validation. Ethical approval was granted by the Bogomolets National Medical University Institutional Review Board (Protocol No. 196, 23/06/2025), with waived informed consent for the use of anonymized retrospective data.

The study period spanned a critical regulatory transition defined by three distinct phases. During the pre-AMS period (2018), practice was guided by the now-repealed MOH Order No. 502, which allowed for broad antibiotic selection (including third-generation cephalosporins) and permitted extended postoperative prophylaxis for ‘conditionally clean’ procedures. In the transitional period (2020), our institution proactively implemented a local stewardship bundle ahead of national reforms. This multi-modal strategy combined educational seminars for surgical teams and monthly audit-and-feedback cycles with structural changes. Specific interventions included the introduction of mandatory ‘stop-orders’ in anaesthesia charts to prevent postoperative dosing and strict pharmacy dispensing controls that restricted ceftriaxone use without specific justification. The post-guideline period (2023) operated under the new MOH Order No. 822, which strictly harmonized national standards with EU guidelines, mandating single-dose cefazolin prophylaxis and prohibiting administration after the surgical incision.

A 3-point compliance score was developed to provide a quantitative measure of adherence to PAP guidelines, awarding one point for each of the following criteria: (1) timing (administration of an antibiotic within the recommended 30–60 min before skin incision), (2) choice (selection of a first-generation cephalosporin [cefazolin] as the primary agent for clean and clean-contaminated procedures), and (3) duration (avoidance of unjustified postoperative antibiotic continuation beyond a single dose for prophylaxis). The primary outcome was the time to discharge, measured in days from surgery.

Statistical analysis was performed using R (v4.3.1, R Foundation for Statistical Computing, Vienna, Austria) with the ‘survival’, ‘MatchIt’, and ‘brms’ packages. Descriptive statistics were used to characterize the cohort and trends over time. To assess the association between the compliance score and time to discharge, a multi-variable Cox proportional hazards model was used, adjusting for the year of surgery, procedure type, and urgency (elective vs urgent). To investigate the stability and reliability of our findings, we conducted a comprehensive series of sensitivity analyses. First, we re-evaluated the primary outcome using a binary definition of adherence (fully compliant [score: 3] vs non-compliant [score 0–2]) to test whether the effect of strict protocol fidelity was masked by partial compliance. Other analyses included a leave-one-year-out analysis and a quasi-causal analysis using propensity score matching with exact matching on procedure type. Finally, a Bayesian hierarchical model with year as a random intercept was fitted to provide a probabilistic estimate of the effect. A *P* value <0.05 was considered statistically significant for frequentist models.

## Results

The distribution of procedure types was stable across the study periods, with laparotomy being the most common surgery (50% overall) (Table I). We observed a significant decrease in the mean LOS from 9.9 (standard deviation [SD]: 9.5) days in the pre-AMS period (2018) to 4.7 (SD: 4.3) days in the post-guideline period (2023) (*P* < 0.001).

**Table I:** Cohort characteristics and main outcomes by period

Characteristic	Overall *N* = 474^a^	Pre-AMS (2018) *N* = 160^a^	Transitional (2020) *N* = 154^a^	Post-guideline (2023) *N* = 160^a^	*P* value^b^
Procedure type					>0.9
Hysteroscopy	116 (24%)	34 (21%)	40 (26%)	42 (26%)	
Laparoscopy	122 (26%)	46 (29%)	36 (23%)	40 (25%)	
Laparotomy	236 (50%)	80 (50%)	78 (51%)	78 (49%)	
Urgency					0.022
Elective	402 (85%)	150 (94%)	122 (79%)	130 (81%)	
Urgent	72 (15%)	10 (6.3%)	32 (21%)	30 (19%)	
Length of stay, days	6.9 (6.9)	9.9 (9.5)	6.1 (4.3)	4.7 (4.3)	<0.001
Compliance score (0–3)					<0.001
0	72 (15%)	44 (28%)	28 (18%)	0 (0%)	
1	118 (25%)	78 (48%)	42 (27%)	0 (0%)	
2	78 (21%)	22 (14%)	30 (19%)	46 (29%)	
3	186 (39%)	18 (11%)	54 (35%)	114 (71%)	

AMS, antimicrobial stewardship; SD, standard deviation.

a *N* (%); mean (SD).

b Pearson’s Chi-squared test; Kruskal–Wallis rank sum test.

**Figure 1:**
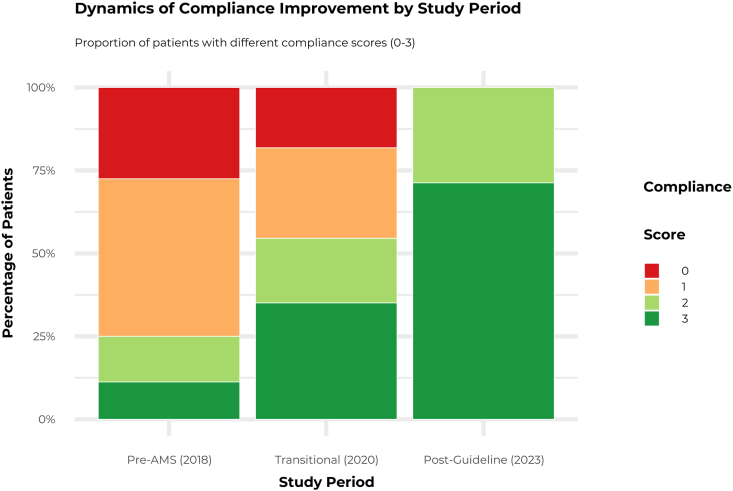
Dynamics of compliance with peri-operative antibiotic prophylaxis guidelines. The stacked bar chart illustrates the proportion of patients within each study period (pre-AMS, 2018; transitional, 2020; post-guideline, 2023) achieving different levels of the 3-point compliance score. The score was calculated based on adherence to guidelines for antibiotic timing, choice, and duration. A score of 3 represents full compliance. AMS, antimicrobial stewardship.

Adherence to all components of PAP guidelines improved markedly over the study period. Timely antibiotic administration surged from low levels in 2018 to 95% in laparoscopy and over 66% in laparotomy and hysteroscopy by 2023 (*P* < 0.001). Guideline-discordant ceftriaxone use was replaced by cefazolin as the dominant agent (90% adoption). Postoperative prophylaxis, administered to 26% of patients in 2018, was eliminated by 2023 (*P* < 0.001). Consequently, the proportion of patients with a low overall compliance score (0 or 1) decreased from 76% in 2018 to 0% in 2023, while the proportion of patients with a full compliance score of 3 increased from 11% to 71% (Figure 1).

Component-level analysis revealed a shift in non-compliance patterns. In 2018, improper timing was the dominant failure (96.2% violation rate), reflecting the ambiguous timing windows of older regulations. By 2023, timing violations fell to 10.0% and duration violations were completely eliminated (0%). However, errors in antibiotic choice persisted as the primary residual challenge (18.8% violation rate in 2023), indicating that prescribing habits regarding specific agents were more resistant to change than process measures.

**Figure 2:**
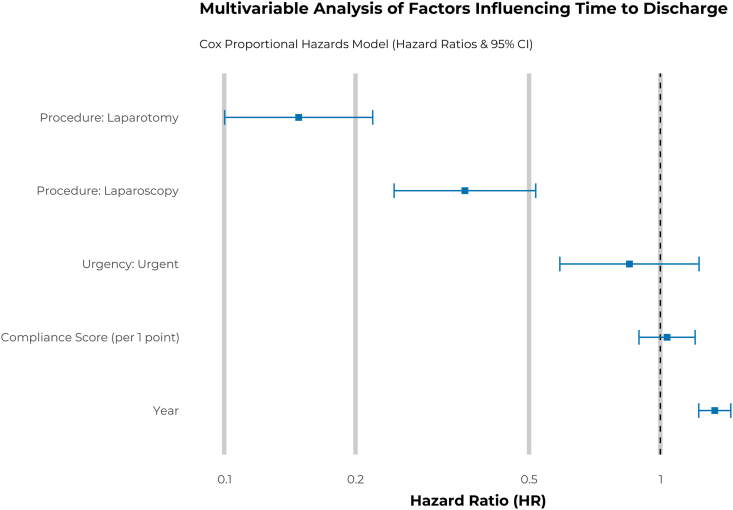
Multi-variable Cox proportional hazards model for time to discharge. The forest plot displays hazard ratios (HRs) and their 95% confidence intervals (CIs) for key predictors. An HR >1 indicates a faster time to discharge (shorter length of stay), while an HR <1 indicates a slower time to discharge. The model was adjusted for all variables shown. Reference groups were Hysteroscopy for Procedure and Elective for Urgency.

In the primary multi-variable Cox proportional hazards model, the year of surgery was the strongest predictor of earlier discharge (hazard ratio [HR]: 1.33 per year; 95% confidence interval [CI]: 1.22–1.45; *P* < 0.001). After adjusting for year and other confounders, the compliance score was not a statistically significant independent predictor of time to discharge (HR: 1.04; 95% CI: 0.89–1.20; *P* = 0.6) (Figure 2). This finding was supported by the Bayesian hierarchical model, which showed a marginally significant result, with a credible interval that narrowly included zero, suggesting a potential small protective effect that did not reach full statistical certainty (estimate: −0.05, 95% CI: [-0.12, 0.01]). Sensitivity analysis using a binary definition of adherence (fully compliant vs non-compliant) showed a trend towards significance (HR: 0.64; 95% CI: 0.38–1.09; *P* = 0.098), though this effect remained statistically subordinate to the year of surgery.

A subgroup analysis revealed a significant interaction with the type of procedure. While the effect of the compliance score was not significant for hysteroscopy or laparotomy, it was a strong and statistically significant predictor of earlier discharge for patients undergoing laparoscopy (*P* = 0.021) (Table II). The leave-one-year-out sensitivity analysis demonstrated the model’s instability concerning the time variable: the effect of the compliance score became statistically significant but changed direction when individual years were excluded, confirming strong confounding by time.

**Table II:** Effect of compliance score in subgroups by procedure type

Procedure	HR	95% CI	*P* value
Hysteroscopy	1.30	0.87, 1.95	0.2
Laparoscopy	1.41	1.05, 1.89	0.021
Laparotomy	1.05	0.86, 1.28	0.6

HR, hazard ratio; CI, confidence interval.

## Discussion

This study demonstrates the significant impact of a multi-faceted AMS program, reinforced by national guidelines, on PAP practices in a Ukrainian maternity hospital. We observed a near-total transformation in adherence, with compliance rates in 2023 surpassing the 60–80% levels commonly reported in other transitional health systems [6]. This improvement was parallelled by a significant reduction in postoperative LOS.

Our most critical finding, however, is the nature of the association between compliance and outcomes. While binary compliance showed a trend towards significance (*P* = 0.098) in our sensitivity analysis, the ‘year of surgery’ remained the dominant predictor of LOS (HR: 1.40, *P* < 0.001). This aligns with the ‘rising-tide’ phenomenon in quality improvement, suggesting that reduced hospitalization was driven by holistic secular trends, including improved sterile techniques, administrative efficiency, and the rigorous hygiene measures introduced during the COVID-19 pandemic, rather than by antibiotic prophylaxis alone [7].

The divergence in component adherence highlights the hierarchy of intervention effectiveness. The complete elimination of duration errors seems to reflect the success of ‘forcing functions’ (such as administrative stop-orders and pharmacy restrictions), which effectively shifted the default bias towards discontinuation. In contrast, the persistence of errors in antibiotic choice (preferring broad-spectrum agents) likely illustrates the challenge of prescribing etiquette, where individual cognitive habits are harder to de-implement than administrative processes.

The significant positive effect of compliance within the laparoscopy subgroup is a noteworthy exception. This may be because laparoscopy is a highly standardized procedure with a more predictable clinical course. In such a controlled environment, the marginal gains from full protocol adherence, particularly timing and antibiotic choice, are more pronounced and less likely to be overshadowed by the greater variability inherent in more complex procedures like laparotomy. This aligns with observations that protocol adherence often fractures under the clinical pressure and patient complexity typical of major open surgeries [8].

This study has limitations, including its retrospective, single-centre design, which may limit generalizability. Furthermore, we were unable to account for all potential clinical confounders that may have influenced the LOS.

In conclusion, institutional and national stewardship initiatives can successfully optimize PAP practices. Our experience offers a replicable model for transitional health systems, demonstrating that stewardship success requires not just resources but also fidelity to core principles: protocol simplicity, actionable feedback, and clinician engagement, a truth also observed in other resource-constrained settings [9,10]. While the overall reduction in LOS is attributable to broad, systemic improvements over time, adherence to specific PAP protocols remains a crucial indicator of quality care and is particularly impactful for patients undergoing highly standardized surgical procedures like laparoscopy. Future efforts should focus on sustaining these high adherence rates and further investigating procedure-specific determinants of outcomes.

## CRediT authorship contribution statement

**O. Pechak:** Writing – original draft, Methodology, Investigation, Formal analysis, Conceptualization. **K. Bielka:** Writing – review & editing, Supervision, Resources, Project administration. **M. Frank:** Writing – review & editing. **H. Fomina:** Writing – review & editing. **D. Yevstifeiev:** Writing – review & editing, Validation, Software. **L. Yanitska:** Writing – review & editing, Validation, Supervision.

## Ethics statement

Ethical approval was granted by the Commission on Bioethical Expertise and Ethics of Scientific Research of the Bogomolets National Medical University (Protocol No. 196, dated June 23, 2025).

## Funding sources

There are none to declare.

## Conflict of interest statement

None declared.
